# The Control of a Hospital

**Published:** 1919-03-29

**Authors:** 


### THE CONTROL OF A HOSPITAL.

On March 29 the subscribers to the Enfield Cottage Hospital in annual meeting assembled will be called upon, in effect, to adjudicate the dispute between the governors and the medical staff (see The Hospital, February 8, 1919, p. 397). The latest move in the controversy is the submission of a joint letter of resignation by sixteen out of the eighteen governors, including the Chairman, the Honorary Secretary, and the Honorary Treasurer. In taking this course, which was practically the only alternative to dismissing the whole of the medical staff, the sixteen governors appear to have been actuated by a desire not to interrupt the service of the hospital. The subscribers can either accept these resignations, which implies that they must find a new committee prepared to take on the management in harmony with the medical staff and to accept the responsibility for collecting the necessary funds; or they must prevail upon the sixteen to resume office, which will be notice to the doctors that they are to obey the rules or be replaced. Both sides in these unfortunate disputes seem to have made certain blunders, and tact in dealing with their opponents has been distinctly to seek. The trouble in its inception, so far as can be made out from a study of the *ex parte* statements contained in letters from the opposing factions to the local press, was a difference of opinion, seven years ago, between the governors and one of the medical staff as to the use of certain brands of ether and chloroform. Moved by the emphatic pronouncements of (the late) Sir Frederic Hewitt, which were noted and echoed at the time in this journal, the governors sought to introduce in the interests of economy the use of these anæsthetics made from duty-free materials, and costing, therefore, about a third of what was previously in use. In this desire the governors, in our judgment, acted within their plain rights; and this appears to have been recognised by the action of the medical staff at the time. Now, however, one of the medical staff, who has been repeatedly at loggerheads with the governors, has refused to use the less expensive material and has induced his colleagues to join in his attitude. His published statements on this purely technical question are so wild and reckless that it is not easy to understand how he has managed to get his colleagues to back him up; and the suspicion is aroused that the governors must have played their hand very badly to induce a body of professional men to go to extremes thus. Other causes of friction have also arisen, especially over the admission of patients; and the medical staff have flatly refused to obey the rules of the hospital in this respect. When their attention is called to the fact, they reply by a request for a joint committee to revise the rules! If the rules are really as antiquated as the doctors suggest, surely their correct course was to get them amended, not to break them deliberately and defiantly; and if the governors declined to move in the matter, to appeal to the annual subscribers' meeting. To that meeting the decision is now referred; but the question has been overlaid with so many personalities and side-issues that there is some danger of the principle involved becoming obscured. That principle, apart altogether from questions of tact and of tactics, is simply whether the lay board of a voluntary hospital, duly appointed by the subscribers, shall or shall not accept the dictation of the medical staff in matters of discipline and of economy. So long as that principle is not lost sight of, the subscribers should have little difficulty in determining their course of action.

